# Cold thermopeaking-induced drift of nase *Chondrostoma nasus* larvae

**DOI:** 10.1007/s00027-023-00955-x

**Published:** 2023-03-24

**Authors:** D. Mameri, D. S. Hayes, S. Führer, E. Fauchery, S. Schmutz, A. Monserat, T. Hasler, D. R. M. Graf, J. M. Santos, M. T. Ferreira, S. Auer

**Affiliations:** 1 Forest Research Centre (CEF) and Associate Laboratory TERRA, School of Agriculture, University of Lisbon, Tapada da Ajuda, 1349-017 Lisbon, Portugal; 2 University of Natural Resources and Life Sciences, Vienna, Department of Water, Atmosphere and Environment, Institute of Hydrobiology and Aquatic Ecosystem Management, Gregor-Mendel-Straße 33, 1180 Wien, Austria; 3 Graduate School of Engineering, University of Tours, 64 Avenue Jean Portalis, 37200 Tours, France; 4 École Nationale Supérieure de l’Energie, l’Eau et l’Environnement, 21 Avenue Des Martyrs, 38031 Grenoble, France

**Keywords:** Thermal fluctuations, Young-of-the-year, Cyprinids, Hydropower, Flume experiments, Pulsed flows

## Abstract

Research on how intermittent water releases from hydropower plants affect the early life stages of fish has advanced in the last years, focusing not only on the direct impacts of rapid flow changes (hydropeaking), but also on the short-term fluctuations in water temperature (thermopeaking). Flow and thermal fluctuations caused by hydropeaking may affect fish movement patterns and migration at critical stages of a species’ life cycle, e.g., by inducing passive downstream drift. Using two experimental outdoor channels, we investigated how nase (*Chondrostoma nasus*, Cypriniformes) larvae respond to a rapid drop in water temperature during hydropeaking (simulating a cold thermopeaking event), reaching on average 5.5 °C under peak flow (maximum discharge) conditions, in comparison with a hydropeaking treatment with a constant water temperature regime. Responses of fish larvae were analyzed during acclimation, up-ramping (increase in discharge), peak flow and down-ramping (decrease in discharge) phases. Fish drift increased during peak flow in the cold thermopeaking treatment compared to hydropeaking. Higher drift rates were also negatively associated with pronounced water temperature drops during peak flow conditions. In addition, the starting temperature of the experiment influenced drift during up-ramping. Overall, the results suggest that cold thermopeaking may increase drift in the early life stages of cypriniform fish compared with hydropeaking with stable water temperature. Hence, monitoring and active water temperature adjustments following hydropower releases should be adopted as strategies to mitigate power plant-related impacts on aquatic organisms.

**Supplementary Information:**

The online version contains supplementary material available at 10.1007/s00027-023-00955-x.

## Introduction

Throughout their life cycle, fish shift between habitats for feeding, reproduction, and sheltering (Lucas and Baras 2001). Drift, which can be defined as a downstream movement of aquatic organisms, willingly (active) or forced by water velocities exceeding a species’ swimming capability (passive), plays a fundamental role in the migration of early life stages of fish such as larvae (Zens et al. 2018; Nagel et al. 2021). Drift has long been described as an important fish migration process to search for more suitable rearing habitats (Jonsson 1991; Lucas and Baras 2001; Reichard et al. 2004; Pavlov et al. 2008; Koster et al. 2013; Lechner et al. 2016). While fish swimming performance can determine the success of habitat shifts, these movements are triggered by changes in environmental conditions. Particularly flow velocity and water temperature are known to be among the main environmental drivers of fish migration in search of more suitable areas for each life stage (Jonsson 1991; Poff et al. 1997; Caissie 2006; Rakowitz et al. 2008; Webb et al. 2008; Olden and Naiman 2010).

Humans impact flow velocity and water temperature through the construction of river regulation infrastructure, of which hydropower plants are one of the most common worldwide (Steel and Lange 2007; Shen and Diplas 2010; Toffolon et al. 2010; Jones and Petreman 2014; Couto and Olden 2018; Hayes et al. 2018; Song et al. 2018). Hydropeaking is one operation mode of hydropower plants, consisting of short-term flow fluctuations downstream of dams caused by the rapid release of water from turbines due to peaks in energy demands (Greimel et al. 2018; Hayes et al. 2022a). Research on hydropeaking increased significantly in the last decade (Boavida et al. 2015; Auer et al. 2017; Romão et al. 2018; Costa et al. 2019; Amaral et al. 2021; Hayes et al. 2021; Führer et al. 2022). Particularly for early life stages of fish, such as larvae and juveniles, the impacts of hydropeaking can endanger successful recruitment and, ultimately, their survival, as it may cause fish to become stranded or passively drift, making them unable to reach critical habitats for life cycle requirements (Kupren et al. 2011; Rolls et al. 2013; Wang et al. 2013; Lechner et al. 2018).

More recently, research on the ecological impacts of hydropeaking focused not only on the direct impacts of rapid flow changes, but also on the associated short-term fluctuations in water temperature (Carolli et al. 2011; Bruno et al. 2012; Schülting et al. 2016; Choi and Choi 2018; Feng et al. 2018; Auer et al. 2023), a process known as thermopeaking (Zolezzi et al. 2010). Thermopeaking occurs due to water stratification in reservoirs, following a seasonal pattern (McCartney 2009; Toffolon et al. 2010; Hayes et al. 2022a). When releases from deeper water layers in stratified reservoirs occur (hypolimnetic discharges), it may lead to a temperature drop in the receiving river (cold thermopeaking), particularly during the summer season. In alpine rivers, water temperature during peaking operations can cool down the water temperature downstream the dam up to 6 °C in spring and summer (Zolezzi et al. 2010). Contrastingly, in winter, the opposite pattern is observed, with an increase in temperature in the receiving river—warm thermopeaking (Zolezzi et al. 2010).

While awareness of the impacts of thermopeaking is growing, its ecological impacts on freshwater populations are still poorly understood. Much of the published literature on the ecological effects of thermopeaking focuses on macroinvertebrate drift (e.g., Carolli et al. 2011; Bruno et al. 2012; Schülting et al. 2016). Fewer studies have assessed the impacts of thermopeaking on fishes (Auer et al. 2023; Casas-Mulet et al. 2016). Understanding the impacts of abrupt changes in temperature due to thermopeaking is particularly important, considering that it can lead to involuntary downstream displacement, i.e., passive drift (Young et al. 2011; Auer et al. 2017, 2023). Such involuntary movements are likely linked to increased hydraulic stress (Fuiman and Batty 1997; von Herbing 2002). Also, fish may seek areas with more optimal temperatures, entailing habitat shifts (Keckeis et al. 1997; Schiemer et al. 2002; Auer et al. 2023).

Therefore, it is crucial to understand how fish cope with rapid changes in flow conditions and associated short-term thermal variations caused by hydropower activity to establish the best mitigation frameworks, including active temperature adjustment of the water released during hydropeaking. In this study, we evaluated the impact of hydropeaking and cold thermopeaking on larvae of nase *Chondrostoma nasus* (L.), a cypriniform species for the conservation of European rivers, whose populations have been declining in the last several decades (Jurajda 1995; Schiemer et al. 2002; Hayes et al 2022b). We performed flume experiments in an outdoor semi-natural stream facility to assess whether cold thermopeaking could lead to a higher fish drift than hydropeaking without temperature changes. We predicted that (1) hydropeaking with cold water release (cold thermopeaking) would entail greater fish larvae drift than hydropeaking with constant water temperature, and that (2) the more pronounced the temperature drop, the higher the drift will be, and (3) fish drift will occur not only in higher velocity areas, but also closer to the shoreline (Auer et al. 2023).

## Material and methods

### Experimental set-up

This study was conducted in the summer, from July 22nd until August 3rd, 2021. All trials were conducted during daylight, from 8:30 to 18:30, in the absence of rain. We used nase larvae (mean TL = 25.1 mm ± 2.1 SD) from a fish hatchery in Lower Austria that used wild-caught breeders (Auer et al. 2017). Larvae were transferred to the HyTEC (Hydromorphology and Temperature Experimental Channel) facility in Lunz am See (Lower Austria) and reared in circular holding tanks (with an approximate volume of 0.7 m^3^ each) 1 month before the start of the experiments. The tanks had a continuous water supply from lake Lunzer See and fish were fed 2–4 times a day with live brine shrimp (*Artemia*) at different hours each day to avoid learning effects (Brodersen et al. 2008). Larvae were in the sixth (VI) larval stage of development (Penaz 1974), with a total length ranging from 20.9 to 29.2 mm at the start of the experiments.

**Fig. 1:**
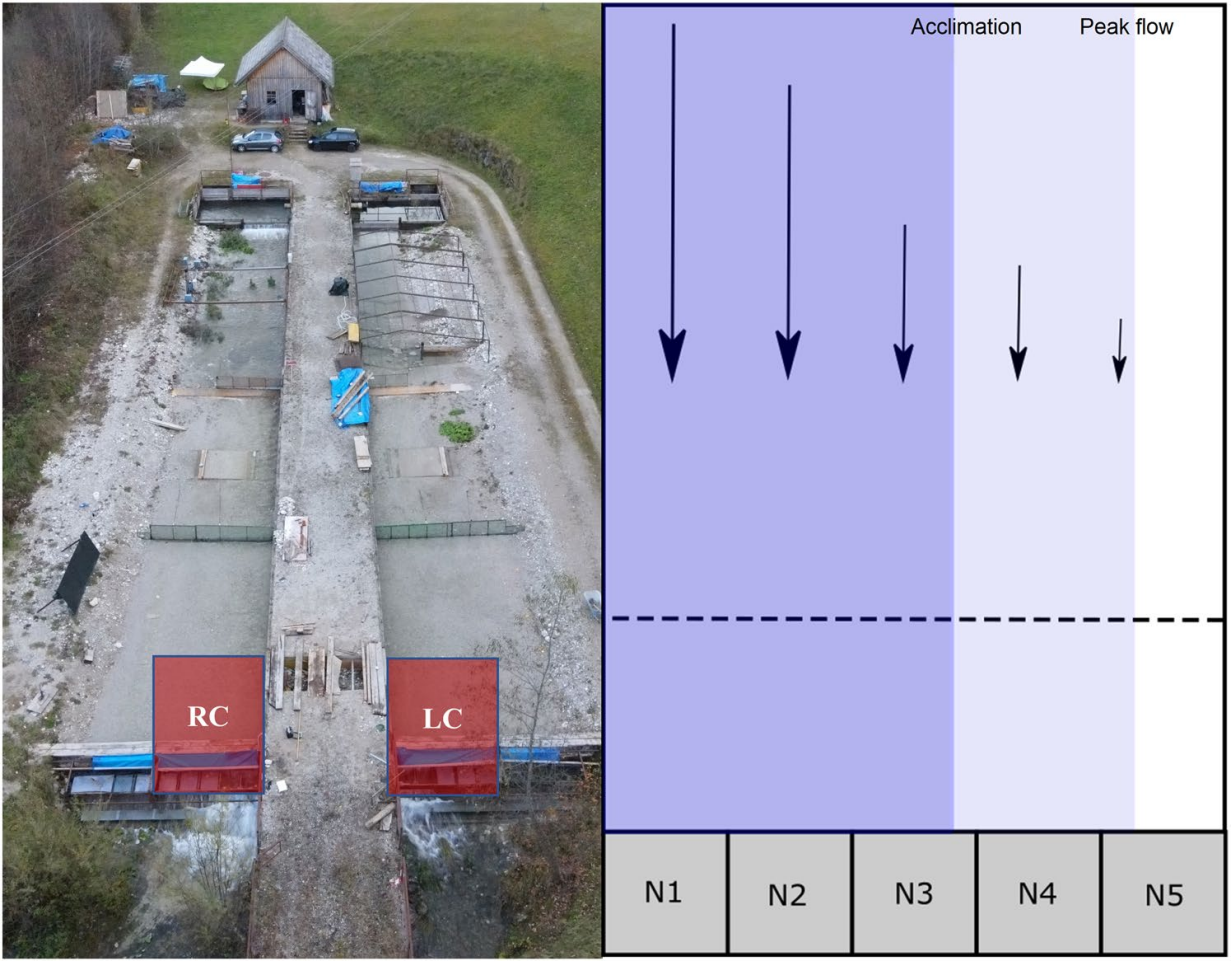
(Left) Aerial picture of the experimental facility (https://hydropeaking.boku.ac.at). The lower sections of both channels (RC–right channel, LC–left channel, both in red) were used for the trials. (Right) Scheme of the experimental area in LC (symmetrical to the one in RC): flow direction (and velocity) is indicated by the (length of) arrows, and the downstream drift nets (64 × 75 cm) are numbered from the deepest and fastest segment (N1) to the shallowest and slowest segment (N5); the wetted area at acclimation (wetted width: 1.50 m) and peak flow (2.60 m) are represented by the blue and light blue fills, respectively; the dashed line refers to the cross-section where flow velocity was measured (Fig. 2)

**Fig. 2:**
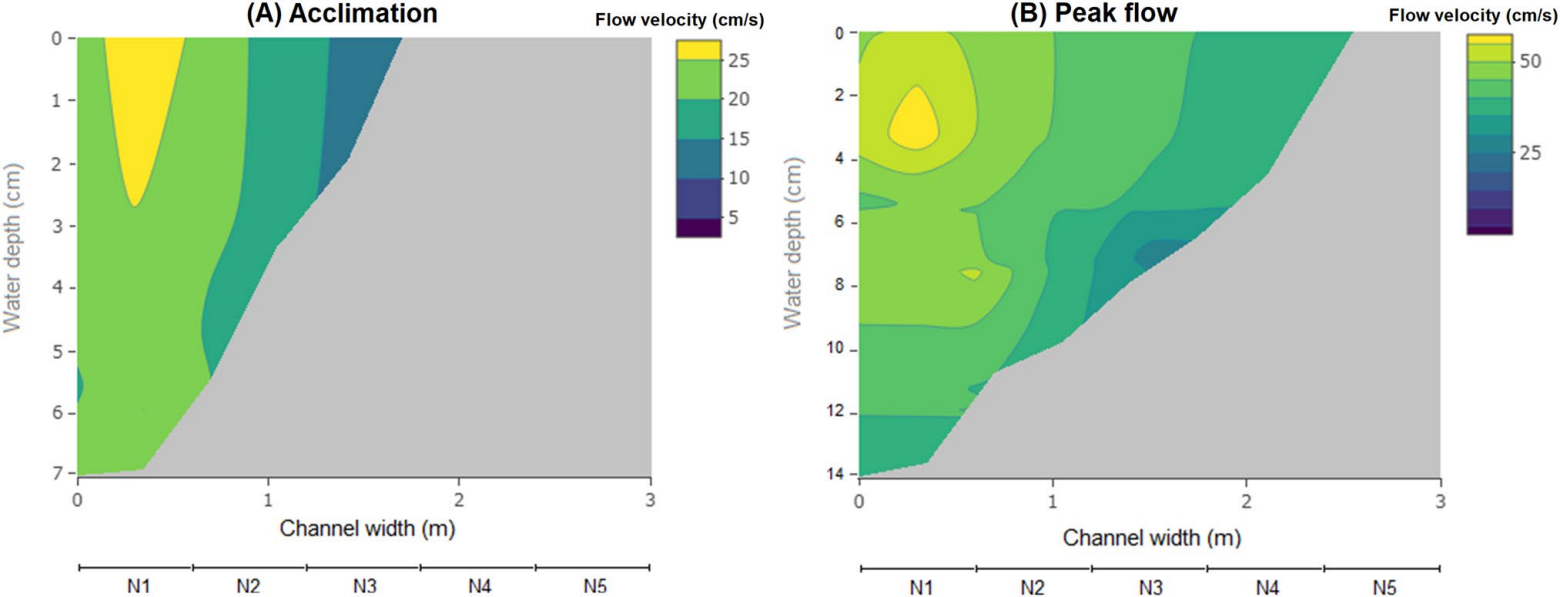
Channel hydraulics during **A** acclimation (15 L·s^−1^) and **B** peak flow (80 L·s^−1^). Water depth (*y*-axis, in cm) and flow velocity (contour, in cm·s^−1^) were measured every 30 cm along a cross section (Fig. 1), from the deepest part of the channel to the shoreline (*x*-axis), with up to three measurements alongside the water column in each point coordinate. The color legend is relative to each plot. The area without water (including the gravel bank with a lateral slope of 5%) is represented in grey. Nets range from N1 (fastest and deepest segment) to N5 (slowest and shallowest)

The experimental facility consists of two outdoor semi-natural channels, 40 m long and 6 m wide, a research station for controlling the water discharge in the two channels, and two pressure pipelines that take the required water from Lunzer See and transport it to the channels (Fig. 1). One pipe is located near the surface and the other is installed deeper, allowing temperature manipulation in both channels and fish tanks. The water is led back into Lunzer Seebach, the lake’s natural run-off, via a height-adjustable dam beam construction at the downstream end of the experimental channels. Channel water temperature, depth, and flow velocity are easily adjusted, guaranteeing controlled and repeatable experimental conditions (Auer et al. 2023; Haug et al. 2022). The two parallel channels had a longitudinal slope of 0.5% and a lateral slope of 5%, with a flat bank substrate dominated by fine gravel and coarse sand (median grain size *d*_50_ = 2.0 mm, 90th percentile grain size *d*_90_ = 5.1 mm). The experimental area encompassed the most downstream section of both channels (4 m length and 3 m width), with five drift nets installed at the lower end of each channel (Fig. 1). Flow velocity measurements (Flowtherm NT–Hoentzsch) were conducted immediately before the start of this study and checked on both channels during the experimental period to ensure identical flow velocity gradients. Water depth and flow velocity were measured every 30 cm along a cross section, with up to three measurements alongside the water column in each point coordinate (Fig. 2).

**Fig. 3:**
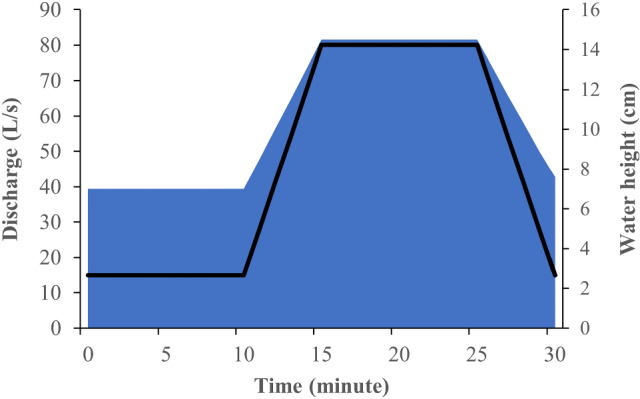
Overview of the experimental setup: channel discharge, in L·s^−1^ (black line) and water height, in cm (blue area) throughout each trial (total duration of each trial: 30 min)

**Fig. 4:**
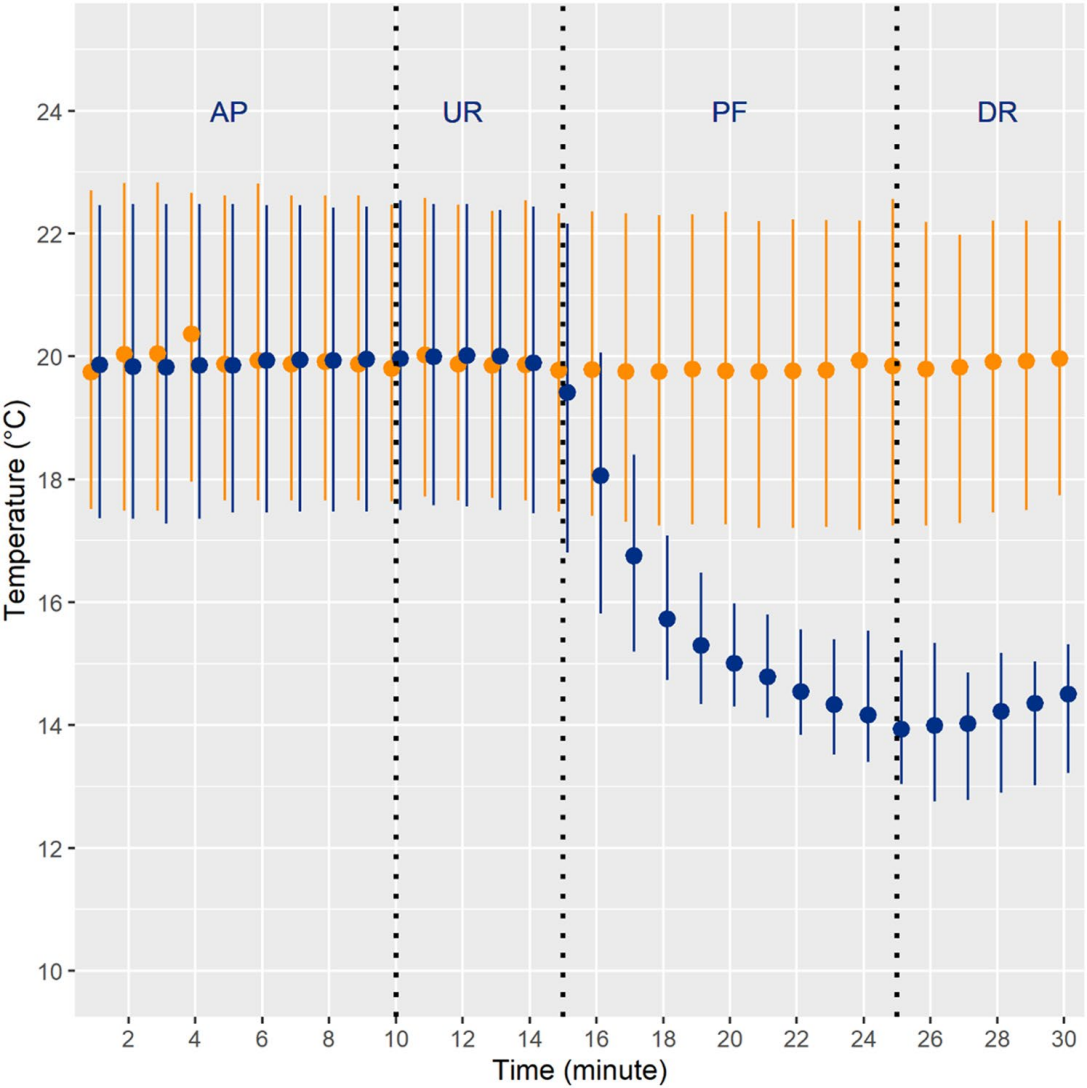
Temperature measurements during hydropeaking (orange) and cold thermopeaking (blue) experiments (1-min time resolution), for each phase of a trial: acclimation phase (AP), up-ramping (UR), peak flow (PF), and down-ramping (DR). Dots and whiskers represent the mean values and 95% confidence intervals, respectively. At the start of up-ramping (minute 10), there was a water temperature drop in the cold thermopeaking treatment, but not in hydropeaking (where temperature remained constant)

Two hydropeaking treatments were implemented: one with a constant temperature regime (“hydropeaking”) and a second one where the temperature dropped rapidly during hydropeaking (“cold thermopeaking”). Each trial lasted 30 min, starting with 50 nase larvae being transported in opaque buckets and stocked in the upstream channel section (3 m ahead from the drift nets and 1 m behind the upper limit of the trial area) at a base flow of 15 L·s^−1^. The water temperature in the tanks (mean = 20.0 °C ± 1.6 SD) and channels (mean = 19.8 °C ± 1.7 SD) was nearly identical, with an average temperature difference of 0.3 °C. Also, the starting temperature in hydropeaking (mean = 19.8 °C ± 1.8 SD) and cold thermopeaking (mean = 19.8 °C ± 1.7 SD) trials was the same. Each experiment consisted of four phases, simulating a single-peak hydropeaking event in Alpine rivers facing flow regulation by hydropower plants (adapted from Auer et al. 2017): (1) a 10-min acclimation period at baseflow, (2) up-ramping, with a discharge increase during 5 min, with a vertical up-ramping rate of 1.5 cm·min^−1^, (3) peak flow, with a discharge of 80 L·s^−1^ that was maintained for 10 min, and (4) a 5-min down-ramping, resulting in a vertical down-ramping rate of 1.4 cm·min^−1^ (Fig. 3). In each phase, we counted the fish that drifted into the nets at the downstream end of the channel (Fig. 1). After each trial, all remaining larvae were cleared from the channel at base flow using hand nets. Overall, nine replicates were performed for each treatment. Drift was compared between the two different treatments (hydropeaking versus cold thermopeaking) for each phase of the trials (acclimation, up-ramping, peak flow, and down-ramping); additionally, comparisons in drift among the different nets (N1–N4) were also performed (Auer et al. 2023). The temperature was recorded on a minute basis using a multiparametric probe (Flowtherm NT–Hoentzsch), complemented with values recorded using pressure probes (Aquitronic ATP05). During cold thermopeaking, water temperature started dropping during up-ramping, reaching its maximum drop during peak flow [mean drop ± SD = 5.5 ± 1.7 °C compared with hydropeaking (Fig. 4].

### Data analysis

Larvae drift, expressed as drift rates, was obtained by dividing the absolute frequency of drifted fish (per phase, considering all nets) by the absolute frequency of fish present in the channel at the beginning of each phase (i.e., that did not drift in previous phases) (Auer et al. 2017):

$${\text{Drift}}\;{\text{rate}} = \frac{{{\text{frequency}}\;{\text{of}}\;{\text{drifted}}\;{\text{fish}}}}{{{\text{frequency}}\;{\text{of}}\;{\text{fish}}\;{\text{in}}\;{\text{the}}\;{\text{channel}}}}.$$

Data normality and homoscedasticity were assessed using the Shapiro–Wilk and *F* test of equality of variances, respectively. As both assumptions were not met, comparisons of drift between different experimental treatments (hydropeaking and cold thermopeaking) were performed with the non-parametric Mann–Whitney test. Differences among trial phases and lateral drift distribution [in which net fish were found (Fig. 1)] were evaluated using the non-parametric Kruskal–Wallis test (followed by Dunn post hoc tests for pairwise comparisons).

Effects of hydropeaking and thermopeaking on fish drift were further explored through multiple regression models, following a stepwise approach where the starting temperature of each trial and the temperature drop at peak flow were included as predictors for fish drift in each phase, considering all trials pooled together (*n* = 18). The “drop1” function from the “stats” package (R Core Team 2021), which uses the Akaike Information Criterion (AIC), a weight of evidence approach, was used to obtain the most parsimonious model (with the lowest AIC), with the thermal variables most associated with fish drift in each phase. Finally, a chi-squared test was performed to assess if the lateral drift distribution (in which net the fish were found) depended on the treatment (hydropeaking and cold thermopeaking). All analyses were conducted in R (version 4.1.0) at a significance level (*α*) of 0.05.

## Results

### Drift during a hydropeaking event

**Fig. 5:**
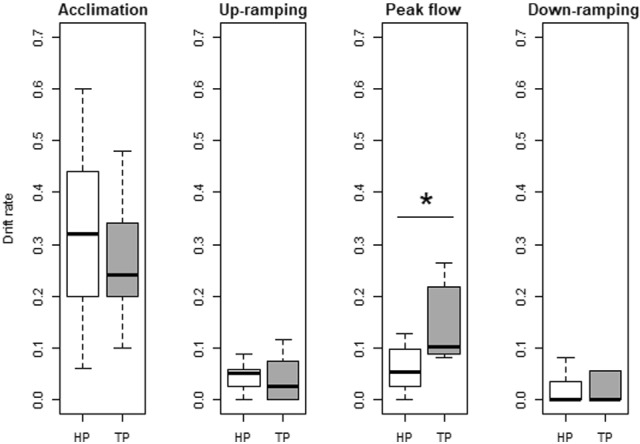
Drift rates of hydropeaking (HP—white) and cold thermopeaking (TP—dark grey) treatments during each of the four phases: acclimation, up-ramping, peak flow, and down-ramping. Bold lines and whiskers outside the boxes refer to median values and interquartile ranges, respectively. Significant differences (*p* < 0.05) in drift rates between HP and TP are marked with “*” (peak flow: *U* = 15, *p* = 0.027)

Drift was significantly different between the phases of the hydropeaking (*χ*^2^ = 19.276, *df* = 3, *p* < 0.001) and the cold thermopeaking treatments (*χ*^2^ = 19.649, *df* = 3, *p* < 0.001). Fish drift was highest during acclimation for both treatments, representing around 30% of the total drift observed in all trials (Fig. 5). For hydropeaking trials, drift during acclimation was significantly higher (mean ± SD = 0.32 ± 0.17) than the ones observed in the subsequent phases (Dunn post hoc, *p* < 0.05): up-ramping (mean ± SD = 0.04 ± 0.03), peak flow (mean ± SD = 0.05 ± 0.05), and down-ramping (mean ± SD = 0.03 ± 0.04). For thermopeaking trials, fish drift at acclimation (mean ± SD = 0.29 ± 0.16) and peak flow (mean ± SD = 0.18 ± 0.15) were not significantly different (Dunn post hoc, *p* = 0.176). However, both were significantly higher than during up-ramping (mean ± SD = 0.05 ± 0.07) and down-ramping (mean ± SD = 0.04 ± 0.07). Comparing the two treatments per phase, results showed that larvae drift in peak flow was higher during cold thermopeaking than during hydropeaking (Mann–Whitney, *U* = 15, *p* = 0.027). These differences were not observed in the other three phases (Fig. 5): acclimation (*U* = 44, *p* = 0.790), up-ramping (*U* = 42, *p* = 0.928), and down-ramping (*U* = 35, *p* = 0.616).

### Temperature influence on drift

An effect of the starting temperature and temperature drop was observed at different phases of the trials. The final adjusted models for each phase retained only one variable each: starting temperature for acclimation and up-ramping, and temperature drop magnitude for the peak flow and down-ramping phase (Table 1). However, significant regressions were only found for drift during up-ramping (with starting temperature as a predictor) and peak flow (with temperature drop as a predictor). For up-ramping, this association was negative: lower (colder) starting temperatures were associated with higher fish drift during up-ramping (*β* = −0.469, *R*^2^ = 0.176, *p* = 0.047). On a similar level, during peak flow, higher temperature drops (colder water) were linked to more pronounced fish drift (*β* = 0.509, *R*^2^ = 0.192, *p* = 0.039).

**Table 1:** Summary of the stepwise regression models for drift rates in each experimental phase (acclimation, up-ramping, peak flow, down-ramping), considering all trials pooled together (*n* = 18)

Phase	Acclimation	Up-ramping	Peak flow	Down-ramping
*Starting temperature*				
*β* coefficient	−0.456	−0.469		
Adjusted *R*^2^	0.159	0.176	–	–
*p* value	0.057	0.047*	–	–
*Temperature drop*				
*β* coefficient			0.569	0.374
Adjusted *R*^2^	–	–	0.192	0.137
*p* value	–	–	0.039*	0.079

The standardized beta coefficient (*β*), adjusted *R*^2^, and *p* value of the variables retained in the best-fitting model (lowest AIC—Akaike Information Criterion) are presented for each phase (significance at *α* = 0.05 marked with an asterisk “*”)

### Lateral drift distribution

**Fig. 6:**
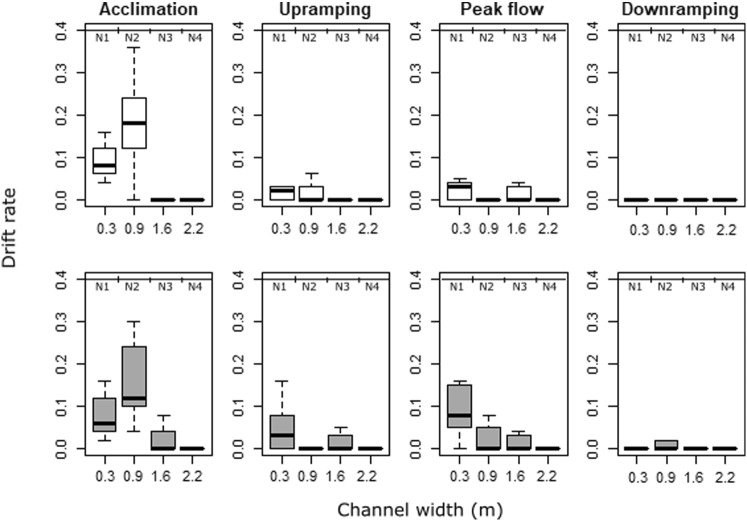
Drift rates at hydropeaking (HP—white) and cold thermopeaking (TP—dark grey) in relation to the distance from the channel (0.3, 0.9, 1.6, and 2.2 correspond to the mid-points of N1, N2, N3 and N4, respectively; no drift was observed in N5), during each of the four phases: acclimation, up-ramping, peak flow, and down-ramping. Bold lines and whiskers refer to median values and interquartile ranges, respectively

Considering all trials pooled together, the majority (90%) of displaced fish were found in N1 (43%) and N2 (47%), located in mid-channel areas of higher flow velocities, reaching up to 58 cm·s^−1^ during peak flow conditions (Fig. 2). Contrastingly, few fish drifted into N3 (9%) and N4 (1%). N5 was only partially wetted during the trials (Fig. 1), even during peak flow, and no fish were found in this net. Significant differences were found in the lateral drift distribution of both treatments, considering all phases (*χ*^2^ = 10.1, *df* = 7, *p* = 0.018). This difference was mainly caused by the observed drift within the first 1.6 m of the channel (N1–N3) during peak flow, where higher drift rates were found for cold thermopeaking fish than for hydropeaking ones (Fig. 6).

## Discussion

### Hydropeaking and thermopeaking impact on drift

The survival of early life stages of fish strongly depends on favorable habitat conditions. Adequate flow and thermal conditions can determine recruitment success and, subsequently, population viability (Lucas and Baras 2001; Sonny et al. 2006; Kupren et al. 2011; Hayes et al. 2021). Hence, it is crucial to understand how early life stages of fish are affected by rapid and artificial variations in flow velocity and water temperature caused by hydropeaking power plants. Such process-based knowledge is needed to evaluate the impacts of hydropeaking and thermopeaking, and to develop adequate mitigation strategies (Schmutz et al. 2015; Sanz-Ronda et al. 2019; Hayes et al. 2022a). By conducting this experimental study, we aimed to assess how hydropeaking and cold thermopeaking affect nase larvae drift.

In both hydropeaking and cold thermopeaking treatments, drift was highest during acclimation. This result is consistent with preliminary trials performed with nase larvae, where the drift was also higher in the first minutes after stocking than the rest of the time (Fig. S1). Hence, this initial period not only allowed fish to acclimate to the experimental set-up under controlled conditions, but also served to remove potentially unfit fish before the start of the experiment (Auer et al. 2017; Mameri et al. 2019). In the hydropeaking treatment, the drift rate did not differ during up-ramping, peak flow, and down-ramping. In contrast, at cold thermopeaking, the drift rate was significantly higher during peak flow (when the temperature change was more pronounced) than during up-ramping and down-ramping. Sudden drops in temperature under rapidly increasing flows, accompanied by increased hydraulic stress, may promote downstream drift, potentially as a behavioral response to maximize successful dispersal (Lechner et al. 2013; Zens et al. 2018), which may be harmful if there is no suitable habitat downstream (as in the case of this experiment, with the drift nets). The results suggest that nase larvae react more strongly to temperature changes than to changes in hydraulic conditions (Zitek et al. 2004; Rolls et al. 2013). Indeed, rapid decreases in water temperature can also lead to “cold shock” in fish and reduced swimming performance (Smith and Hubert 2003; Donaldson et al. 2008). Looking into each phase, peak flow exhibited the greatest temperature drop of the thermopeak (surpassing 5 °C), and higher drift rates were positively associated with the magnitude of the temperature drops during peak flow (occurring in the cold thermopeaking trials).

Swimming performance depends on water temperature, and when facing suboptimum thermal conditions, a fish’s swimming ability may be compromised due to lower levels of oxygen diffusion in the skeletal muscles (Farrell 2002). This phenomenon may have reduced larvae responsiveness to increased flow conditions (Donaldson et al. 2008; Morgan et al. 2022), even though the water temperature during the trials was within the thermal tolerance range of the nase (8.0–29.0 °C; Leuven et al. 2011). This reduced swimming performance is likely to have, at least partially, led to an overall increase in drift rates in cold thermopeaking during peak flow when compared with hydropeaking. It should be noted, however, that only one temperature drop and one ramping rate (velocity at which the water level increases or decreases) were tested in this experiment. Future studies should assess larvae drift across gradients of temperature and water level variation to understand better the impact of these environmental conditions on fish swimming (Auer et al. 2023).

### Lateral drift distribution and active drift

Lateral fish distribution across the drift nets revealed that larvae that experienced cold thermopeaking drifted more than hydropeaking fish in higher velocity areas in the channel (N1–N2) and closer to the shoreline (N3), suggesting that fish may be less active when exposed to temperature drops (Martelo et al. 2013). The increased drift closer to the shoreline (N3) during cold thermopeaking trials, which has also been observed for juvenile grayling *Thymallus thymallus* (L.) (Auer et al. 2023), may indicate that fish were trying to avoid colder temperatures by moving into the shallow zones in search of thermal refugia. In nature, young stages of cypriniform species are also known to shift closer to the shoreline to avoid faster currents (Bodensteiner and Lewis 1994; Copp et al. 2002; Reichard and Jurajda 2004; Lechner et al. 2013; Greimel et al. 2018). However, the flow velocity measurements at base flow conditions were far below the velocity thresholds described for this species. Indeed, the critical flow velocity for nase larvae with 15–25 mm length is estimated to be 4–5 times their corresponding body length (Flore et al. 2001). These critical values were only exceeded during peak flow in the mid-channel section, but not during base flow conditions.

It should be noted that we did not distinguish between active and passive drift. However, some studies have highlighted the importance of active larvae drift in downstream dispersal (Robinson et al. 1998; Reichard et al. 2004; Pavlov et al. 2008; Lechner et al. 2016). Particularly for the nase, a study in the Danube River revealed that larvae were more active during low flow conditions, thus suggesting that the hydraulic conditions could facilitate active dispersal (Lechner et al. 2018). The high drift rates observed during acclimation in both treatments (hydropeaking and cold thermopeaking), as well of base flow trials, therefore, seem to suggest that active drift may have occurred. Further evidence comes from the fact that cold thermopeaking fish drifted more in N3 than in hydropeaking. Behavioral studies in nature-like channels, like the ones presented here, would help to clarify the role of active and passive drift in the total observed drift caused by hydropeaking and thermopeaking.

### Management recommendations

Earlier studies suggested that hydropower releases into rivers should be adapted to avoid peaking during key life cycle periods of aquatic species (Jones and Petreman 2014; Hayes et al. 2019; Moreira et al. 2019). The release of ecological flows, following recent European policies for the Water Framework Directive implementation, can provide an effective mitigation measure to dampen not only flow ramping rates, but also temperature changes during hydropower releases (EU Commission 2015), namely by adapting water releases during hydropeaking that mimic natural temperature fluctuations (Casas-Mulet et al. 2016; Heggenes et al. 2017; Tonolla et al. 2017; Halleraker et al. 2022). According to Zolezzi et al. (2010), in Alpine rivers, thermopeaking can have the same magnitude as the one simulated in this experiment. This can potentially harm fish by causing increased involuntary downstream displacement, ultimately affecting population viability.

In addition to drift, medium and long-term effects of both cold and warm thermopeaking should also be investigated, such as displacement of larvae and early juveniles from the regulated site, food availability, and spawning success in the following reproductive season. Finally, one aspect which was not targeted in this experiment was stranding caused by the fish’s inability to shift from shallow to deeper areas during down-ramping (Führer et al. 2022; Hayes et al. 2023). Conducting similar thermopeaking experiments with a larger variety of microhabitats may help to investigate how temperature fluctuations can promote habitat shifts and lead to larvae drift and stranding (Auer et al. 2017, 2023; Antonetti et al. 2023; Hayes et al. 2023).

## Conclusions

Overall, our results highlight that hydropeaking, combined with cold thermopeaking, increases the drift of sensitive life cycle stages of cypriniform fish populations, if occurring during sensitive life cycle stages. To our knowledge, this was the first experimental study assessing the influence of cold thermopeaking in larvae of a cypriniform species and comparing it with hydropeaking. Hence, ensuring suitable flow and water temperature conditions for early life stages of migratory fish, downstream from hydropower plants, is of utmost importance to avoid involuntary passive drift and ultimately population decline. Monitoring and active adjustment of water temperature following intermittent water releases should be included as main mitigation strategies to establish best-practice hydropower operations.

## Supplementary Information

Below is the link to the electronic supplementary material.

Supplementary file1 (PDF 31 kb)

## Data Availability

Supplementary data is provided alongside this manuscript. Generated data from this study is available from the corresponding author upon reasonable request.
