# Supplementary material for: Cold thermopeaking-induced drift of nase Chondrostoma nasus larvae
Source: Aquat Sci. 2023 Mar 24;85(2):56. doi: 10.1007/s00027-023-00955-x (PMC10038962; doi:10.1007/s00027-023-00955-x)
Supplement: Supplementary file 1 — Supplementary file1 (PDF 31 kb) [file 27_2023_955_MOESM1_ESM.pdf]

**Supplementary Material**

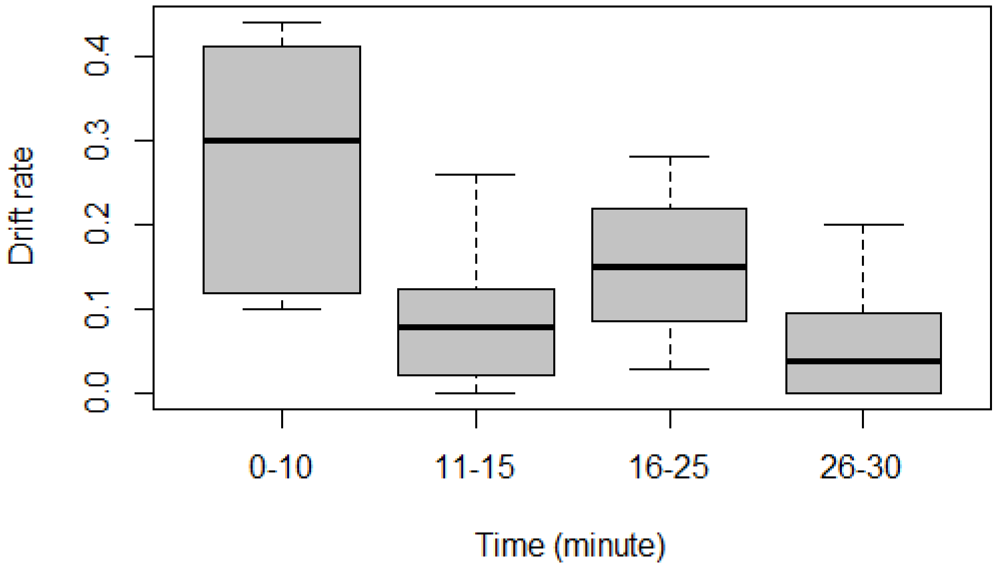


**Fig. S1** Drift rates in base flow trials (50 fish stocked in each), with a constant discharge of 15 L $s^{-1}$ for 30 minutes. Drift rates were calculated for the time periods corresponding to each of the experimental phases: acclimation (minutes 0-10), up-ramping (11-15), peak flow (16-25) and down-ramping (26-30). Bold lines and whiskers refer to median values and interquartile ranges, respectively.
